# Historical Account of Gaol Fever

**Published:** 1857-10

**Authors:** Francis C. Webb


### AN HISTORICAL ACCOUNT OF GAOL FEVER.

By FRANCIS C. WEBB, M.D., F.S.A.

[*Read before the Society on Monday, July 6th,* 1857.]

Amongst the successes which have crowned efforts made in the cause of sanitary reform, few are more worthy of remark than the change which has been effected in the physical condition of prisoners and its consequent effect,—the diminution of fever amongst the population of Great Britain. Up to the commencement of the nineteenth century, fever was not only common in gaols, but it had come to be considered their peculiar product, their indigenous and appropriate growth. As might be supposed, the mischief was not confined to its source, and "gaol distemper," as it was called, was a frequent cause of mortality in our towns and cities. The history of prison fever forms a remarkable page in a retrospect of the physical condition of our countrymen, and its cessation may be considered as one of the noblest triumphs achieved by practical philanthropy. Space will not permit me to review minutely that condition of prisons and their inmates which obtained until the present century was considerably advanced. For information on this subject I can do no more than refer to the writings of Howard, Neild, and Buxton. The appreciable causes which favoured the dissemination of contagious fever in gaols are well known; they were the crowding of human beings in small cells and subterraneous dungeons, the withdrawal of air and water, the want of nourishment and exercise, of clothing and bedding, the filth consequent on the absence of sewerage and necessaries, a sanguinary and ill regulated penal code, and, as the result of all, mental depression which must have amounted, in most instances, to despair.

The following historical notices are offered as a contribution towards a more complete history of the subject. As affording a picture of the past they are of some interest, but they do more than this, they furnish a complete answer to those who question the amount of good accruing to society at large, from enlightened measures for ameliorating the physical condition of its lowest strata.

The earliest historical reference I have found to gaol disease occurs in the year 1414, when, according to Stow, the gaolers in Newgate and Ludgate died, and prisoners in Newgate to the number of sixty-four. Neither in this or in the seven preceding years have I met with any account of epidemic disease, so that whatever the exact nature of this visitation might be, it had evidently acquired its virulence from the locality and its attending circumstances.

The first outbreak of which I have found mention occurred in the year 1522, at Cambridge. Hall, in his *Chronicle*, tells us, that at the assize kept at the castle of Cambridge, in Lent, the justices and all the gentlemen, bailiffs and others, resorting thither, took such an infection, whether it were of the savour of the prisoners or of the filth of the house, that many gentlemen, as Sir John Cut, Sir Giles Alington, knights, and many other honest yeomen, thereof died, and almost all which were there present were sore sick, and narrowly escaped with their lives. I may observe, that when he speaks of the filth of the house, he refers to the castle in which the prisoners were confined, and which was still used as the county gaol in the time of Howard.

1577 was the year of the celebrated black assizes at Oxford. This was, perhaps, the most fatal outbreak of gaol fever of which we have an account. Five hundred and ten persons are said to have died of it, from the 6th of July to the 10th of August. The historians Camden and Stow make particular mention of this occurrence; but the fullest account of it is given by Thomas Cogan, a graduate in medicine of the University of Oxford, in an Appendix to his *Hauen of Health*, published in the year 1586. It appears from these memorials, that a large concourse of people was assembled at the assize held in the castle of Oxford on the 4th of July, 1577, on the occasion of the trial of one Rowland Jenkes, whom Camden styles a saucy, foul mouthed bookseller, and who had spoken injuriously of the queen. His trial created a considerable sensation at the period, and there were many of the county gentlemen and townspeople present. Suddenly a pestilential disease appeared amongst them. Those present referred it to various causes, some to a poisonous and pestilent vapour, some to the stink of the prisoners, some to the damp of the ground, and some to the poisonous and pestilent breath of the unfortunate Jenkes. However, there cannot be a doubt, from the evidence, that it was a most fatal form of continued fever brought into the court by the prisoners, and this is confirmed by the testimony of Lord Bacon. The jurors died presently, struck down by the poison. They were followed by the two judges, Sir Robert Bell, Lord Chief Baron, Serjeant Baram, Doile, the high sheriff, five of the justices, four councillors at law, and an attorney. Three hundred died in the town of Oxford, and upwards of two hundred more in the neighbouring country. No women or children are said to have been carried off; and it is remarkable that down to a late period this was held to be a peculiarity in the ravages of the gaol distemper. From the account which Cogan gives of the symptoms, it is evident that it was an adynamic form of fever, accompanied by an altered condition of blood as evidenced by hæmorrhages and petechiæ ("sanguine spots in many parts of the body.") He expressly tells us that depletion by bloodletting and purging was ill borne. When Howard visited Oxford, he was informed by the gaoler that, some years before, wanting to build, he obtained the stones for the purpose from the ruins of the Castle Court. On digging, he found a skeleton with fetters on the legs, probably one of the malefactors who had died in court at the black assize of 1577.

Two years after this occurrence, in the March of 1579, a petition was presented to Queen Elizabeth on the subject of the condition of the prisoners in the Queen's Bench Prison. From the circumstance that double the usual number had been confined there, a disease had broken out which went by the expressive name of "the sickness of the house." During the preceding six years near an hundred persons had died of it, and many were still sick and in danger of their lives. The author of the *Survey of London* tells us "that the disease was engendered chiefly or rather only of the small and few rooms in respect of the many persons abiding in them, and these, by want of air, breathing in one another's faces as they lay, which could not but breed infection, especially when any infectious person was removed from other prisons thither."

In 1585 gaol distemper was rife in Exeter gaol. It broke out at the assizes, and was most fatal to those in court. Out of the twelve persons composing one jury, eleven died of it.

I have been hitherto unable to extract from the records of the seventeenth century any particular instances of disease occurring in prisons. Not that we can suppose it to have been absent, for during the great civil struggle pestilential fever spread its ravages amongst the prisoners of war, the forces in garrison, and the armies in the field. I need only refer to its ravages at Reading in 1643, in the army of the Earl of Essex in the same year, at Newark before it was relieved by Prince Rupert, and at Winchester and Bristol in 1645. But we are unable to separate from these and similar accounts the exact part played by the prison element. One reason of the paucity of detail yielded by this period is to be found in the frequent epidemics of plague. Sydenham treats of plague and pestilential fever in connection, and there is no doubt that most cases of petechial fever were confounded by ordinary observers with the true pestis. Another reason is furnished by the great political excitement of the time. Historians were so occupied in chronicling the stirring events which were taking place around them, that they probably neglected the less exciting, though not less useful, records of the silent progress of disease. It is not, however, improbable that notices of occurrences bearing on our subject are to be found, although we have hitherto failed in discovering them. The matter is certainly worthy a more extended research.

Never were the prisons of England in a worse condition than during the eighteenth century. War and peace alternated; and each cessation of hostility, flooding the gaols with the refuse of our fleets and armies, was consequently marked by outbursts of gaol distemper. On the other hand, at each gaol delivery the recruiting sergeant, standing at the door of the prison, sought candidates for the soldier's laurel amongst the liberated inmates. We shall hereafter find, on good authority, how constant a source was here of mortality to our sea and land forces. Again, though administered with what in the present age seems unnecessary barbarity, never were the laws worse obeyed than during the reign of the first Georges. On any reference to this period, the mind at once reverts to the scenes painted by Hogarth, and described by Gay and Fielding. But we need not trespass on the provinces of the novelist and dramatist for what is preserved in the sober page of history. I would only refer to the account which Smollet gives of the prevalence of crime in 1730, a year marked by the death of one of England's ablest judges through the infection of gaol fever. His description obtains ample confirmation from the report of the Monthly London Sessions for April in that year, to be found in the *Political State of Great Britain.*

Consequent on the crowding of prisons produced by this state of public morals, gaol fever broke out most severely in the western districts. According to one account, some prisoners, who were brought from Ivelchester gaol to be tried at the Lent assizes at Taunton, infected the court. Lord Chief Baron Pengelley, Sir James Sheppard, serjeant-at-law, and John Pigott, Esq., high sheriff, died, besides many of the inferior class. The writer in the *Political State of Great Britain,* however, appears to state that the outbreak took place at Exeter. He says, "also Mr. Serjeant Rous, who attended at the assizes at Exeter, and was taken ill there as the others were, but being not so very bad at first, he hastened to London, but died in a few days after he came to his own house." The same authority states, that there were above a hundred prisoners tried, many of them said to be sick of the gaol fever. It is highly probable that the disease was present both at Exeter and Taunton.

The years 1740 and 1741 were marked by famine and epidemics of dysentery and fever in Ireland. The penal institutions may therefore be considered as only participating in the general ills of the country. Still the following facts are illustrative of our subject. On the 10th of March, 1741, a report was made by Dr. Philips to Baron Wainwright upon the state of the prisoners in Limerick gaol. It shows that a great number had died during the previous week of dysentery, and that of the remainder many of them were in a very miserable condition for want of subsistence, others not likely to live, and many very ill with a malignant fever. The judge died of the fever after his return to Dublin from the Munster circuit. The gaol at Tralee was so full that there was not room for the prisoners to lie down; fifty died in six weeks time, and above a hundred were tried, mostly for stealing the means of sustenance.

Dr. Huxham has left us an account of a very fatal petechial fever which devastated the neighbourhood of Launceston in the year 1742. It was of a thoroughly adynamic type, presenting in a most marked degree all the symptoms of typhus. He asserts that it had its origin in the prisons, and was disseminated far and near by the county assizes.

One of the most remarkable notices of an outbreak of gaol fever is given by Sir John Pringle. It occurred in the year following the last rising in the Stuart cause, 1746. About the middle of May in that year, the army under the command of the Duke of Cumberland was encamped at Inverness. They were joined by Brigadier Houghton's regiment, which, with three more, landed at Nairn as a reinforcement. A few days after, twelve men of that corps were sent into the hospital with fever of a low type, which did not admit of depletory treatment. On inquiry, he found that this disease had been contracted in the following manner. A few months before, he says, we had taken a French ship, on board of which were some troops sent to assist the rebels, and in that number some English soldiers, who having formerly deserted to the French in Flanders, as malefactors, were, on their arrival in England, thrown into gaols; where they were kept till an opportunity offered of sending them by these transports to be tried by a court-martial at Inverness. These prisoners, thirty-six in number, brought with them the gaol distemper, and gave it to the regiment with which they were embarked. In three days after coming ashore, six of the officers were seized with it; and the regiment, in the few days it lay at Nairn, left about eighty sick; and in the ten following that it remained at Inverness, one hundred and twenty, ill of the same fever, were sent into hospital. He elsewhere informs us, that from the hospitals it spread amongst the inhabitants of the town.

The peace of Aix la Chapelle was concluded in October, 1749, and as a consequence a great part of the forces were disbanded. From the difficulty of obtaining employment, and doubtless in many cases from aversion to labour, numbers embraced a life of crime. The result was that the swarming prisons were devastated by the fever. At the Old Bailey Sessions, in May 1750, it broke out: many were infected, and about forty of the upper class and many of the lower, who were present, died. The names of twenty are given in the *Gentleman's Magazine* for that year. Amongst them were Sir Samuel Pennant, the lord mayor, Sir Daniel Lambert, Baron Clark, of the Exchequer, and Mr. Cox, the under sheriff. A hundred prisoners underwent their trial on this occasion. They were kept in the bail dock, and two other small rooms, measuring 14 feet by 11, during the several days occupied by the sessions. It is curious that the contagion principally attacked those who occupied the side of the court to the left of the Lord Mayor; thus the Middlesex jury, who were on the left, lost many, whilst the London jury, on the right, escaped. The Lord Chief Justice and the Recorder, who sat on the right of the Lord Mayor, took no harm, whilst most of the occupiers of the bench on his left suffered. It was inferred that the stream of air from the window directed the *materies morbi* to that part of the court. With two or three exceptions, all who were seized with the fever died. Some who escaped the distemper were attacked with diarrhœa, which, however, yielded easily to treatment. It is not known what proportion of nurses and attendants of the sick suffered. On the representation of Lord Chief Justice Lee, the city authorities took the state of the prisons into consideration. Newgate and some of the other gaols were ordered to be cleansed and provided with ventilators.

With that deliberation, however, which befits the dignity and usually marks the proceedings of civic functionaries, the ventilation of Newgate was not properly effected for two years afterwards, during which time, in its loathsome cells, six or seven died weekly. In October 1750, Pringle and the Rev. Dr. Hales were consulted as to the best means for procuring purity of air, and preventing infectious disease in Newgate. They advised that a ventilator, worked by a machine in the manner of a windmill, should be erected on the leads. Their advice was accepted, but the plan was not put into execution until July 1752. The erection of this ventilator was marked by decided benefit to the prisoners; the gaol became less offensive and sickly, and although fever was not extinguished, its fatality was much diminished, the deaths falling from six or seven a week to about two a month. In erecting the ventilator eleven workmen were employed in and about Newgate, and of these seven were attacked by the gaol distemper. One of these men conveyed the disorder to six of his family. Sir John Pringle read a most interesting account of these occurrences to the Royal Society in February 1753; his paper appears in the *Philosophical Transactions* for that year. It is curious that more than one of the patients ascribed his attack to the inhalation of the offensive air escaping from the tubes of the old ventilator, which inhalation was immediately followed by nausea, faintness, and violent vomiting.

In 1755, a prisoner from Exeter gaol carried the fever to the town of Axminster. It raged there with considerable violence, and proved fatal to many who were attacked.

In 1761 fever was rife amongst the prisoners of war at Portchester and Winchester. In some of the cases this appears to have presented symptoms not unlike those of the true plague; for buboes in the groins and armpits were observed. It had entirely disappeared at Dr. Lind's inspection in 1762.

In 1772 Warwick county prison was the scene of the ravages of the distemper. It carried off the gaoler and several of the prisoners.

About the same time an outbreak occurred at Bedford. It had been very fatal in the gaol, and the contagion spread to the inhabitants of the town, of whom many died. Mr. Daniel, the surgeon to the gaol, was carried off. His successor changed the treatment from sudorifics to bark and cordials, and a sail ventilator being erected, the fever did not reappear.

Dr. Lettsom, in his *Medical Memoirs*, relates the case of fourteen persons, who were attacked by the same disease in May 1773: they all resided in a court in Long Lane, Aldersgate Street. The infection was brought into the court by a person who had been confined in Newgate, and had there caught the distemper. Several of these people were females; and Lettsom makes the observation, that he believes them to be the first recorded cases of women being attacked by the gaol fever. In this he was wrong: several cases related by Pringle were also of the fair sex. His forty-eighth and forty-ninth cases are two instances of death in Wood Street Compter, from the same cause. The patients were in both cases debtors, fathers of families, and neither of them owed more than three guineas.

In 1773-74, fever raged in the prison at Aylesbury.

In 1775, Howard found in Dublin Newgate numbers ill with gaol distemper, unattended and disregarded. In 1776, he mentions that he saw cases, and there were several deaths from it, at Hertford. He relates the incident, that a prisoner was brought out as dead from one of the dungeons, but, on being put under the pump, shewed signs of life, and soon after recovered.

In 1781, an extraordinary and very fatal epidemic of fever occurred amongst the Spanish prisoners of war at Winchester. Sir James Carmichael Smith, who has left a full account of this visitation, states that it appeared to have been brought from on board the Spanish ships. It decidedly was not the same disease that was prevalent in English prisons; it differed especially in the absence of petechiæ and marked head symptoms.

In the borough and county gaols, the fever was now apparently rapidly dying out; in 1779, Howard only saw one case, and that in Newgate. In 1782, he did not find a single case in the whole kingdom. This amelioration was no doubt greatly due to his own exertions. But in January 1783, preliminary articles of peace were signed at Versailles between England, France, and Spain. The next month the militia was disbanded, and the forces were otherwise reduced. Again the prisons became crowded, and again the gaol fever appeared. It was at this date that it was so fatal at Worcester. From thence it was carried to Droitwich by a prisoner, who infected his own family and many of the inhabitants. Fourteen died there in a short time.

That the prisons were constant sources of disease to our army and navy during the last century, finds ample confirmation in the writings of Lind and Pringle. The former says,

"the sources of infection to our armies and fleets are undoubtedly the gaols. I have had many patients under my care at Haslar Hospital, particularly deserters from the Marines, who were very ill of the distemper, and brought it immediately from thence." Elsewhere, he tells us that the seeds of this fever were carried by the fleets to all quarters of the world, particularly to North America; and that it occasioned more mortality amongst the seamen, than all other diseases and means of death put together.

The transportation of felons was also a means of disseminating the malady. Our penal settlements, which, until the year 1774, were situated in America, frequently suffered from its introduction.

I would terminate this historical sketch by a few remarks on the nature of the gaol distemper of the eighteenth century. I need, perhaps, scarcely say, that it was typhus fever in its most developed form. It was characterised by the rapid pulse, the loss of strength, the anxiety and depression, the dry black tongue, the critical days, the head symptoms; by the low delirium, with red ferret-like eye, or by coma; by the absence of marked abdominal symptoms until the last stage, when involuntary dark offensive evacuations took place; by the offensive character of the secretion from the skin; and always, in a considerable proportion of cases, by the eruption we now call the mulberry rash, running on into vibices and purple petechiæ; by the tendency to hæmorrhage; and, in many instances, by early dissolution,—the *typhus siderans* of authors. If in some cases the early stages presented what appeared to be an inflammatory condition, a depletion never failed to sink them into the lowest state of exhaustion. It was the same disease as the hospital and ship fever, identical with the pestilential fever of Sydenham. Our retrospect, therefore, throws some light on the etiology of typhus. We know not, perhaps may never know, what the *materies morbi* of typhus may be; but we do know that it invariably appeared in our prisons when human beings were crowded together, living in their own filth, without air, food, and water, and subjected to every depressing influence. Again, from the materials here collected, an argument is fairly deducible against the concentration of fever poison by collecting cases in a circumscribed area. It matters not whether it be within the walls of a gaol, or of a fever hospital or ward; the teaching of recent experience is on this point in accordance with the results of historic investigation.

Lastly, every fact in our review bears its testimony to the contagious character of typhus. The men who saw it in a form and prevailing to an extent rarely equalled in more modern times, were unanimous in their conclusion; and few can read the unvarnished narratives of Pringle, Lind, Huxham, and Lettsom, without acknowledging its justice.

As the peculiar denizen of our prisons, fever has now disappeared; the gaol distemper no longer holds a place in scientific treatises, or in popular apprehension; and already, probably, few who attend our courts of justice recognise, in the rue thickly strewn in the prisoners' dock, the evidence of the dread which was once inspired by the unfortunate occupants.

